# Navigating into the unknown: exploring the experience of exposure to prehospital emergency stressors: a sequential explanatory mixed-methods

**DOI:** 10.1186/s12873-023-00906-7

**Published:** 2023-11-15

**Authors:** Ali Afshari, Mohammad Torabi, Sasan Navkhasi, Marzieh Aslani, Afshin Khazaei

**Affiliations:** 1 Department of Medical Surgical Nursing, Nursing and Midwifery School, Hamadan University of Medical Sciences, Hamadan, Iran; 2 Department of Nursing, Malayer School of Nursing, Hamadan University of Medical Sciences, Hamadan, Iran; 3 Department of Prehospital Emergency Medicine, Asadabad School of Medical Sciences, Asadabad, Iran; 4 Instructor of Critical Care Nursing, Department of Nursing, Asadabad School of Medical Sciences, Asadabad, Iran

**Keywords:** PTSD, Emergency medical technicians, Psychiatric disorders, Prehospital emergency care

## Abstract

**Introduction:**

The unpredictability of prehospital emergencies combined with constantly changing circumstances can lead to increased stress and mental health issues among Emergency Medical Technicians (EMTs). To accurately determine the stress-inducing factors in the prehospital environment, it is important to first identify the stressful events that occur in this environment. Therefore, this study strives to provide a thorough analysis of the stressors in the prehospital environment.

**Methods:**

Sequential explanatory mixed methods were conducted in Hamadan prehospital emergency centers in 2022. The study included 251 EMTs, who were selected through a method in the quantitative phase. The quantitative part used a questionnaire consisting of basic information and the Posttraumatic Stress Questionnaire (PCL-5). In the qualitative phase, 17 with extensive experience in dealing with prehospital stressors were selected based on their PCL-5 scores (above 33). The qualitative phase analysis was carried out using the contractual content method using the Graneheim and Ladman's approach. Statistical analyzes for the quantitative and qualitative phases were performed using SPSS 21 and maxqda 10, respectively.

**Results:**

The study revealed that the EMTs had an average PTSD score of 21. 60 ± 11. 45. Multivariate linear regression analysis showed that the number of shifts had a statistically significant relationship with PTSD scores (t = 26.38, *P* < 0.001). The qualitative phase of the study included 17 interviews, resulting in 14 subcategories, which consisted of four categories: “the overall impact of the stress crisis on technicians,” “missing links in the communication network in incident management,” "professional shortcomings in pre-hospital care,” and “the complex and multifaceted context of stressful pre-hospital emergencies.” Additionally, the study's theme was centered around “surveying the experiences of EMTs in stressful environments.”

**Conclusion:**

As the number of shifts increased, the primary cause of the high prevalence of PTSD in EMTs was revealed. Prehospital emergency stress can be reduced and managed more skillfully by adjusting various factors such as shortening workdays, offering continuous training, augmenting workforce, supplying ambulance equipment insurance, refraining from hiring personnel devoid of clinical training, hiring psychologists, hiring midwives in an emergency, updating prehospital protocols and guidelines, encouraging cooperation between EMTs and other relief groups, and utilizing cutting-edge technologies.

## Introduction

Emergency Medical Technicians (EMTs) play a critical role in providing medical care to critically ill patients. However, frequent exposure to stressful events can have significant physical, mental, and psychological impacts on healthcare providers working in prehospital settings [1–4]. Changes in an individual's physiology and behavior can be caused by stressors, which are stimuli that disrupt the body's balance and trigger a stress reaction. stressor are stimuli that disrupt the body's balance and trigger a stress response, which can lead to changes in an individual’s physiology and behavior [5, 6]. The unpredictable of emergencies in the prehospital settings, along with the dynamic environment, can lead to stress and psychological problems in EMTs [3, 7–11]. EMTs ability to perform well in planned scenarios could be affected by increased levels of anxiety, according to a study of their performance under simulated high-stress conditions. Additionally, there is the potential to impact medication calculations and reduce documentation. Furthermore, it can impair medication calculations and reduce documentation [12, 13]. Hagiwara and his team showed in their results that, under certain circumstances, emergency technicians can make errors that could potentially lead to patient death in approximately 4.3 out of 100 emergency operations [14].

Prehospital environments are known to contain stressors such as seeing someone die, having clients physically attack you, having threats made with weapons, seeing colleagues threatened, being exposed to toxic substances, having serious auto accidents, and dealing with pediatric, obstetric, and gynecological emergencies [15–18]. In pre-hospital emergencies, appropriate techniques are required for precise stressor diagnosis, identification, and explanation. The majority of research on stress has mostly employed quantitative techniques and methodologies [19–21]. Nonetheless, a thorough knowledge of the experiences and viewpoints of EMTs in managing stress remains lacking. Owing to the psychological and mental aspects of stress, a multifaceted strategy involving qualitative research techniques is imperative [22, 23]. Thus, there are still notably few studies that specifically address stressors in prehospital emergencies, despite significant research efforts in this area [24–26]. This knowledge gap emphasizes how much more qualitative research in this field is required. Recognizing the need for mixed methods in this field, the goal of this study is to close the knowledge gap and offer a more thorough analysis of stressors in the prehospital setting. This study's primary goals were to ascertain the prevalence of post-traumatic stress disorder (PTSD) EMTs and to pinpoint risk factors. 2) Use quantitative data to investigate and clarify the experiences of stressors in prehospital emergencies.

## Methods

### Study design

A sequential explanatory mixed-method approach was employed in this study to achieve the research objectives. The initial data collection process consisted of collecting quantitative data, followed by an analysis of the data, and then using the results to collect qualitative data The initial data collection process included the collection of quantitative data, followed by an analysis of the data and the use of the results to further collect qualitative data [27]. Because of the importance of the qualitative part of the research in achieving the research objectives, more weight was given to the qualitative part than to the quantitative part. The reason for using the quantitative part was used at the beginning of the research to obtain an information-rich sample and purposeful selection of participants to enter the qualitative part (participant selection model) and to help design or modify qualitative interview questions [28]. The research project was approved by the Research Ethics Committee of the Asadabad School of Medical Sciences (ethical code IR. ASAUMS. REC.1402.021). All methods were performed in accordance with the guidelines and regulations of the Ethics Committee.

### Settings and participants

#### Quantitative study

The research population for the quantitative study was made up of EMTs who were chosen by census method and worked in emergency bases in Hamadan city (2022). However, the sample size formula was used in a limited population to determine the appropriate number of participants to achieve an acceptable power (80%). This was done by taking into account a relative error of 10% (r = 10%) and a confidence interval of 95% (1-α/2 = 95%), as well as a 22% prevalence of PTSD (*p* = 22%) found in the study by Iranmanesh et al. As a result, a sample size estimate of 251 people (n) was determined. It should be mentioned that 307 operational technicians were working in the Hamadan emergency bases at the time of the study [29].

$${n}_{0}=\frac{{Z}_{1-\frac{\alpha }{2}}^{2}\left(P\right)\left(1-P\right)}{{\left(rp\right)}^{2}}$$

$$n=\frac{n}{1+\frac{{n}_{0}}{307}}$$

n0: sample size in an infinite population, n: Sample size in a limited population, p: prevalence of PTSD, r: relative error

The criteria for participation in the quantitative study included operational EMT, willingness to participate, more than one year of work experience, and no known neurological or mental illness. In addition, EMTs who had experienced stress outside of work, such as the death of a loved one, in the previous 8 weeks were excluded from the study. In total, 259 EMTs were included in the quantitative analysis

#### Data collection

The research tool in the quantitative part included a questionnaire with basic information consisting of two parts:1) demographic information, including age, work experience, educational qualification (nurse, emergency medical technician, operating room technician, anesthesia technician), education (postgraduate, bachelor's, postgraduate, and higher), type of employment (formal, contract, contract, plan), marital status (married, single, separated), base location (city, road, air), number of missions, and number of shifts in the last month); And 2) the Post-Traumatic Stress Questionnaire (PCL-5), a 20-item self-report instrument (on a 5-point Likert scale) designed in 2013 by Weathers et al. developed in accordance with the new DSM-5 criteria, which aims to screen and preliminarily determine the rate of PTSD in EMTs [30].

After the necessary explanations for completing the questionnaires, the researcher gave the technicians basic information and the PCL-5 questionnaires and asked the participants to complete the first part (demographic characteristics) and then the PCL-5 checklist, after which they were given to the researcher. In the quantitative part of this study, the important objective was to identify EMTs with PTSD based on the scores obtained in the qualitative sample selection of the study.

#### Validity and reliability

Regarding the properties of the instrument (validity and reliability), numerous studies have been conducted in most countries, and almost all studies have indicated the validity and reliability of the PCL-5 for detecting PTSD [31–34]. The adequacy of the Persian version of this instrument was also evaluated by Sadeghi et al. who reported satisfactory validity and reliability (α = 90.7) of this instrument in identifying individuals with PTSD [30]. In the current study, the dependability of the PCL-5 total score, as measured by the Cronbach's alpha coefficient, was 89%. For each aspect of aggressive thoughts, avoidance, negative changes in mood or cognition, and changes in arousal and irritability were observed (87%, 88%, 91%, and 90%, respectively), indicating adequate reliability of the instrument.

### Settings and participants

#### Quantitative study

**Fig. 1:**
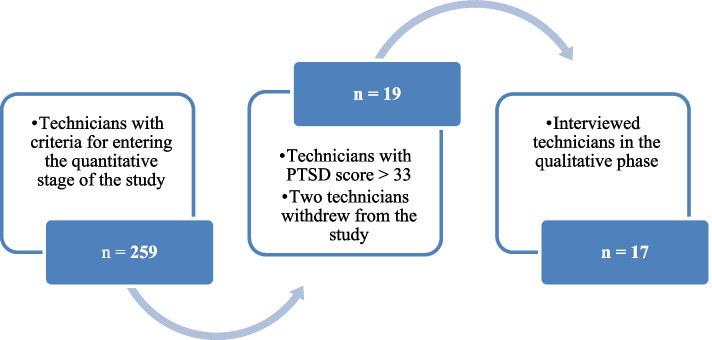
The stages of selecting participants in the quantitative and qualitative stage

Considering that technicians with rich experience in dealing with prehospital emergencies should be identified and included, the sample selection in the qualitative phase was purposeful and based on the results of the instrument used in this phase. Therefore, a score of 33 was established as the optimal cutoff point for the transient diagnosis of PTSD in technicians [35, 36]. Based on the results of PCL-5, 19 technicians with a score above 33 were selected, of which 17 were interviewed (Fig. 1).

#### Data collection

Qualitative data were collected through semi-structured interviews and face-to-face conversations with the corresponding author, who had 20 years of professional experience in pre-hospital care. The interview questions were designed by a team consisting of three assistant professors of nursing with pre-hospital experience and one assistant professor of clinical psychology. The interview guide included questions related to the research objectives, including describing a stressful workday and identifying specific missions that cause stress (Table 1). The interviews were conducted in the training base and in a separate room outside of the technicians' working hours to ensure their privacy and confidentiality. The interviews lasted between 40 and 60 min, depending on the condition and willingness of the interviewee.

**Table 1:** Qualitative phase interview questions

Main Questions	Probes
1. Can you describe your experience of exposure to prehospital emergency stressors as an emergency medical technician? 2. What are some of the most challenging situations you have encountered in your work as an emergency medical technician? 3. How do you cope with the emotional and physical demands of your job? 4. Can you describe a time when you felt particularly overwhelmed or stressed on the job? 5. What challenges do you face from the time you announce the mission to the time you arrive at the scene of the accident? 6. What factors stress you the most when you are at the scene of the accident? 7. Which pre-hospital emergency do you experience the most stress? 8. How do you feel your experiences of exposure to prehospital emergency stressors have impacted you personally and professionally? 9. Can you describe any support or resources that have been helpful in managing the challenges of your job? 10. How do you feel your experiences of exposure to prehospital emergency stressors have impacted your relationships with colleagues, friends, and family? 11. Can you describe any changes you have noticed in yourself since beginning your work as an emergency medical technician? 12. How do you feel your experiences of exposure to prehospital emergency stressors have impacted your overall job satisfaction? 13. Can you describe any specific moments or experiences that have been particularly meaningful or rewarding in your work as an emergency medical technician?	• Can you provide more detail about that experience? • How did you feel in at that moment? • Can you describe any physical or emotional symptoms you experienced in response to that stressor? • Can you provide an example of a time when you felt supported in managing the challenges of your job? • Can you describe any specific strategies you use to cope with the demands of your job? • Can you provide an example of a time when you felt particularly stress of your work as an emergency medical technician?

#### Trustworthiness

Several measures were taken to ensure the trustworthiness of the data [28]. First, the credibility of the results was ensured by the involvement of three expert supervisors who reviewed and confirmed data collection, analysis, and interpretation. This ensured that the results were valid and reliable. Purposive sampling was used to select participants and key informants with the maximum variation and appropriateness. This approach facilitated the inclusion of diverse viewpoints and ensured the authenticity of the data. To increase dependability, the accuracy of data analysis was verified by three independent researchers with experience in qualitative research who were assistant professors of nursing and clinical psychology. Their expertise and independent review made the analysis more and reduced the risk of bias or errors. Confirmability was ensured by providing transcripts, codes, and categories to the corresponding supervisor, an assistant professor of nursing, and two other faculty members with expertise in qualitative research. Their support of the analysis process adds an external perspective and increases the objectivity of their findings. Finally, transferability was ensured through a detailed explanation of the study characteristics, including the research context, participants, and the process of data collection and analysis. This information enabled further evaluation and assessment of the transferability of the study to other contexts or populations.

By implementing these measures, this study sought to ensure the trustworthiness and rigor of the data, thereby increasing confidence in the results and their potential for future research and practice.

#### Ethical consideration

In quantitative study participants were fully informed about the purpose and benefits of the study and about their voluntary participation. They were also assured that they could withdraw from the study at any time without prejudice. Written informed consent was obtained from all participants. The privacy and confidentiality of participants' data was also maintained through the use of hypothetical codes and names. In quantitative part of study, participants in the interview process were treated with utmost respect and dignity. The study's purpose, interview process, and any potential risks or benefits were thoroughly explained to the participants. They were given the freedom to choose whether or not to participate voluntarily, with the assurance that they could withdraw at any point without facing any negative consequences. Following the interview, a comprehensive debriefing session was conducted to further clarify the study's objectives, address any inquiries, and offer additional support resources if necessary. If any of the questions caused distress to the participants, the interview was stopped and continued with their permission (Table 2).

**Table 2:** Qualified participants in the qualitative phase

**Participant**	**Degree of Education**	**Marital Status**	**Employment Status**	**Work Experience (years)**	**The number of S/M (in the last month)**		**Base location**	**PTSD** **Score**
					**Shift**	**Mission**		
1	1	Single	Contractual	6	13	110	Urban	38
2	2	Single	Planned	2	13	120	Urban	40
3	1	Married	Official	12	7	19	Air	33
4	3	Married	Official	7	13	68	Road	35
5	4	Single	Contractual	6	12	45	Road	33
6	1	Divorced	Official	21	11	37	Air	35
7	5	Married	Official	13	13	105	Urban	33
8	6	Single	Planned	8	14	114	Urban	36
9	2	Single	Contractual	4	13	132	Urban	40
10	2	Married	Planned	7	12	70	Road	38
11	4	Married	Official	12	10	38	Road	36
12	1	Single	Contractual	2	14	130	Urban	42
13	7	Married	Official	21	12	29	Road	37
14	7	Married	Planned	8	13	112	Urban	35
15	4	Married	Contractual	4	14	132	Urban	41
16	6	Single	Contractual	7	13	122	Urban	39
17	8	Married	Official	23	12	8	Air	36

1: Bachelor of Nursing, 2: Associate of emergency medicine, 3: Bachelor of emergency medicine, 4: Associate of operating room, 5: Associate of anesthetist, 6: bachelor of operating room, 7: Master of Nursing, 8: Associate of Nursing

### Data analysis

#### Quantitative data analysis

In our study, we used the Persian version of the PCL-5, which demonstrated strong internal consistency with a Cronbach's alpha of 0.92. To analyze the relationship between the independent variables (demographic factors) and the dependent variable (PCL-5 total score), both binary (univariate) and multivariate linear regression analyses were performed. Multiple linear regression analysis allowed multiple independent variables to be included simultaneously. The best fitting model was choking by examining the accuracy criteria. Collinearity, which refers to the intercorrelation between independent variables, was assessed using the Variance Inflation Factor (VIF). VIF values below 10 and above 0.2 are considered acceptable to avoid collinearity problems. The reliability of the PCL-5 questionnaire was evaluated using the test–retest method. The overall reliability of the questionnaire was 89%. Additionally, the reliabilities for each dimension were 87%, 88%, 91%, and 90%, respectively. These reliability values indicate that the PCL-5 questionnaire is a reliable tool for assessing PTSD symptoms in the present study. All statistical analyses were performed using SPSS version 21, with the significance level set at a 95% confidence interval and a two-tailed *p*-value of less than 0.05.

#### Qualitative data analysis

After collecting and analyzing quantitative data, the study proceeded to the qualitative phase using the contractual content method with Graneheim and Ladman's approach [37]. The analysis units comprised transcribed interviews and observation notes, which were repeatedly read to gain a comprehensive understanding. Semantic units related to the data were summarized and assigned appropriate codes. The obtained codes were compared based on their similarities and differences, and similar codes were placed into categories and subcategories. Finally, themes were extracted by determining the relationships between categories. All coding and analyses were conducted using the MAXQDA10 data analysis software (VERBI Software, Berlin, Germany).

## Results

### Quantitative phase results

The mean age was 6.16 ± 32.79 and the median work experience of the technicians (IQR 5–12) was 9 years. The median number of shifts was 12 (IQR 11–13) and the number of missions per month was 60 (IQR 9–85). In terms of marital status, 83 individuals (32%) were single, 177 were married (66%), and 5 (1.9%) were separated from their spouses. Regarding the employment status, 97 (37.5%) were official, 88 (34%) contractual, 27 (10.4%) planned, and 47 (18.1%) temporary workers. In terms of educational qualifications, 130 technicians (50.2%) had a degree in medical emergencies, 61 (23.6%) had a nursing degree, 35 (13.5%) were operating room technicians, and 33 (12.7%) were anesthesia technicians. The overall mean score of PTSD among EMTs was 21.60 ± 11.45. 24 individuals (20.1%) scored above 33 on PTSD assessment. The mean age of technicians with and without PTSD diagnostic criteria was 6.9 ± 28.88 and 5.54 ± 33.77 years, respectively, and the mean total PTSD score in these groups was 6.08 ± 38.02 and, 7.46 ± 17.47, respectively. The results of the four main dimensions of PCL-5 are shown in detail in Table 3.” Univariate linear regression analysis revealed statistically significant relationships between the independent variables and PTSD scores. Specifically, age (t = -0.195, *P* = 0.02), number of shifts (t = 28.54, *P* < 0.001), number of assignments (t = 4.53, *P* < 0.001), and work experience (t = -6.42, *P* < 0.001) were significantly associated with PTSD scores. After entering all the independent variables in the model and controlling for confounding variables in the multivariate linear regression, variables such as the number of assignments (t = 2.50, *P* = 0.013), work experience (t = -3.24, *P* = 0.001), and the number of shifts (t = 26.38, *P* < 0.001) still had a statistically significant relationship with the dependent variable (PTSD score). Among these, the number of shifts per month was the most important and effective predictor of PTSD score in technicians. Overall, this regression model was acceptable for predicting the value of the dependent variable of the independent variables (F = 279, df = 258, *P* < 0.001), and could explain nearly 77% of the variance in the dependent variable (PCL-5 score).

**Table 3:** Scores of four main dimensions of PCL-5 questionnaire in emergency medical technicians

Dimensions	Number of Item	Mean	Standard Deviation
**Intrusion**	5	4.98	3.08
**Avoidance**	2	2.25	1.70
**Negative alterations in cognitions**	7	7.42	4.63
**Alterations in arousal and reactivity**	6	6.91	4.15

The above table shows that the highest average was related to negative alterations in the cognitive dimension and the lowest average was related to the avoidance dimension

### Qualitative phase results

After conducting 14 interviews, data saturation was achieved [38]. To increase certainty, three additional interviews were conducted, which resulted in no new data. In the qualitative phase, 17 interviews were conducted with qualified participants, resulting in 512 primary codes. After merging and summarizing the codes, 164 codes were identified, leading to 14 subcategories, four categories, and one theme. The study results, including the extracted subcategories, categories, and themes, are summarized in Table 4.

**Table 4:** The extracted subcategories, categories and theme in the qualitative phase

Theme	Categories	Subcategories	Sub/ Subcategories
**Surveying the experiences of EMTs in stressful environments**	**1) The overall impact of the stress crisis on technicians**	**1) Physical effects**	
		**2) Spiritual-psychological-mental effects**	
		**3) Occupational-personal-functional effects**	
	**2) Missing links in communication networks in incident management**	**1) Deficiencies in Inter-organizational Cooperation**	
		**2) Deficiencies in Intra-organizational Cooperation**	
		**3) Deficiencies in External Organizational**	
	**3) Professional shortcomings in pre-hospital care**	**1) Educational Factors**	
		**2) Organizational Factors**	
		**3) Individual Factors**	
		**4) Factors Related to Client and Disease**	
		**5) Contextual Factors**	**1) Moral, Cultural, and Legal Conflicts**
			**2) Environmental Factors**
	**4) The complex and multifaceted context of stressful pre-hospital**	**1) Stressors of pediatric emergencies**	
		**2) Stressors of gynecological emergencies**	
		**3) Stressors of cardiac emergencies**	

## Theme: surveying the experiences of EMTs in stressful environments

### Category 1: the overall impact of the stress crisis on technicians

In this category, participants reported experiencing various signs and symptoms when faced with prehospital emergencies, such as excessive fatigue, aggressiveness, muscle spasms, obsessive thoughts, and decreased self-confidence. This category was further divided into three subcategories: 1) physical effects, 2) spiritual-psychological-mental effects, and 3) occupational-personal-functional effects. Participants provided insightful quotations regarding their experiences and their effects on well-being.

- P5: *“The injured person was under the car and the skull was completely shattered. The scene was very stressful: My colleague had less experience, vomited, and could not stay on the scene. Even the police did not dare to approach.”*
- P6: *“I often sit in a corner of the base due to the stress caused by the mission and think about the terrible scenes of the missions. Sometimes, these stresses have made me forget the simple protocols. Recently, I felt that I was depressed. I don't talk to each other when I'm inside the base.”*

The participants talked about the individual-functional effects that the stressors of their pre-hospital emergency work had on them. These effects included feelings of regret for choosing a job that involved such distressing situations, loss of confidence in sensitive situations, depression brought on by failure in stressful emergencies, and the creation of obsessive thoughts due to performance negligence. Their mental, psychological, and emotional health suffered greatly as a result of these stressors, which in turn had an adverse effect on their regular lives. The stressors technicians encountered while performing their emergency duties had a significant impact on their general mental health and capacity to function.

- P13:* “At the scene of the accident, the child died terribly. This issue had made me mentally disturbed for a long time and I constantly thought about it in my subconscious mind.”*

### Category 2: missing links in communication networks in incident management

Effective communication networks are crucial for seamless coordination and timely response in incident management. However, in the present study, several missing links were identified in different aspects of the communication networks. These missing links can be categorized into three subcategories.

#### Subcategories 1: deficiencies in inter-organizational cooperation

When it comes to incident management, collaboration between various organizations is essential. This includes emergency services, the Red Crescent, law enforcement, and fire departments. Inadequate performance in this domain may result in inaction and delays. The following are some participant remarks that draw attention to these shortcomings:

- P9: *"We were dispatched to a highly critical accident mission. The scene was very crowded. The police arrived at the scene with a significant delay. We were involved in the initial care of patients. Later, we found out that the patient's companions had complained about losing their wallets and valuable belongings."*
- P11: *"The patient was trapped in a car because of an accident, and his clinical condition deteriorated. Unfortunately, fire department personnel arrive at the scene with a significant delay in patient rescue. Eventually, the patient died, and we practically could not do anything."*

#### Subcategories 2: deficiencies in intra-organizational cooperation

Intra-organizational cooperation refers to collaboration within an organization involving different roles, such as technicians, physicians, and operators. The lack of effective communication among these roles can hinder the overall response efficiency. One participant’s comment illustrating this deficiency is as follows.

- P8: *"We took a restless patient to the hospital in less than 3 min, but due to delays in admission and lack of attention, he fell from the stretcher, and in the end, due to the hospital staff's negligence while complaining, our name was on the list."*

#### Subcategories 3: deficiencies in external organizational cooperation

Interactions with patients, companions, and the public are examples of external entities with which an organization may cooperate. Effective communication and cooperation can be hampered by a lack of trust, unfavorable societal perceptions, and high expectations. The shortcomings are further compounded by the relatives of the patients' failure to provide the required care, their refusal to do so, and their refusal to yield to ambulances during periods of high traffic. One participant brought attention to this shortcoming by saying:

- P1: *"Due to the wrong address given to us by the emergency operator, we arrived 15 min late at the scene of a heart patient. The patient's companion insulted us because of the overall delay."*

These deficiencies collectively indicate the absence of appropriate communication networks in the prehospital systems. Addressing these missing links is crucial for enhancing incident management and ensuring efficient response in emergency situations.

### Category 3: professional shortcomings in pre-hospital care

This category consists of sub-categories such as educational factors; individual factors; factors related to the patient and disease; contextual factors, which also include sub-categories called moral, cultural, and legal conflicts; and environmental factors.

#### Subcategory 1: educational factors

Regarding hands-on training in real and simulated settings, the technicians said that they would be far more proficient in carrying out advanced procedures like endotracheal intubation, delivery, and other invasive procedures if they could put what they had learned in theory into practice on a patient. This would be beneficial in lowering anxiety after dealing with a variety of prehospital emergencies. Additionally, technicians suggested that one key way to improve practical skills is to provide lab skills with sophisticated tools and equipment, like mannequin models, that can carry out difficult and invasive procedures. Regarding this, a technician said:

- P10: *"We have not had much retraining; we have passed the same course as the university. Going to the hospital to see and perform natural childbirth has not happened because if we see natural childbirth up close, our stress will be reduced a bit."*

#### Subcategory 2: organizational factors

- The subcategory of organizational stress-reducing factors also includes codes that support technicians by granting leave, intermittent displacement personnel's within urban and road, requiring rest after completing a traumatic mission, minimizing shift work, forming a psychoanalytic team in an emergency, insuring ambulance equipment and gear owned by the organization, dispatching experienced technicians, increasing the number of technicians for trauma incidents, increasing the number of road bases to shorten the time spent on the scene, offering financial and personnel incentives to boost employee motivation, and dispatching doctors to stressful missions alongside technicians.
- P7: *“Our pre-hospital emergency department has worn-out helicopters, which are war-type helicopters, from 50 years ago. It creates a lot of noise and vibrations, so that one cannot focus on patient care. Every time the mission is announced, we will be under a lot of stress because we will go with ourselves and we will return with God.”*

#### Subcategory 3: individual factors

The subcategories of individual stress factors also included codes such as the need for more skill in carrying out the mission and managing the situation, recounting the incident, not focusing and ignoring, practice and study, mental review of the procedures before arriving at the scene, individual use of electronic technologies, refusal to remember stressful scenes, adapting to work conditions, gaining experience and high skills, using research and scientific research to manage stress, thinking about all possible aspects of the disease, and sharing thoughts and experiences. One technician said about dealing with prehospital emergencies:

- P1:* "I became stressed and I am guilty of aggravating my stress. For example, they announce a mission to us, and I feel stressed that I will enter a heartbreaking scene. There is no specific reason, but unfortunately, this happens to me."*

#### Subcategory 4: factors related to client and disease

According to the participants, the sub-category was created based on the patient's medical history and the nature of the illness the client was experiencing. The technicians believed that the presence of these factors contributed to their stress when handling pre-hospital emergencies. This subcategory was created based on codes such as providing patients with medical information, facing violence, stress from the patient's poor state at the accident scene, chronicity or non-emergency of the patient's condition, unpredictable disease outcome, patient refusal to allow pre-hospital procedures, accurate diagnosis of the current problem, and patient unreasonable requests. The following are a few of the analytical units that were gleaned from the technician statements:

- P17: *“We went over the head of a very obese patient with decreased level of consciousness. The patient had no other companions. With a thousand misfortunes and difficulties, we were able to move the patient.”*
- P12:*’ Sometimes we are faced with patients who have relatively high medical information, these patients cause stress. You have to be on the ball, you didn't drop a clanger, or even some people have very high expectations, for example, the patient doesn't have an emergency problem, but he asks us to move him on a stretcher or take him to the hospital.”*

#### Subcategory 5: contextual factors

The EMTs felt that contextual factors, which were also taken into consideration as a subcategory, had a major influence on the development and exacerbation of observable stress when handling pre-hospital emergencies, as reported by the participants. Environmental factors and ethical-cultural-legal conflicts were the two other subcategories that made up this subcategory.

#### Sub/subcategory 1: moral, cultural and legal conflicts

The stress that technicians experienced due to ethical, cultural, and legal factors while carrying out their pre-hospital missions and necessary measures was one of the difficulties that study participants mentioned often. Codes like cultural barriers to job duties, gender limitations in handling pre-hospital emergencies, and a lack of alignment with cultural and ethical duties have given rise to this subcategory. Some of the analysis units in this regard are as follows:

- P14: *“The mother's water bag was ruptured, even the baby's head was in the birth canal, but her husband did not allow me to give birth because of his belief, and I only watched the scene until the patient arrived at the hospital. I was very stressed during this time.”*
- P 13: *“In missions where we encounter patients of the opposite sex, we often have problems, such as examining organs in random cases or even taking blood pressure. For example, in an accident that results in a double fracture of the leg, the area must be exposed so that the bleeding can be stopped or a splint can be placed on the patient. This is because the patient's companions do not allow us to perform the procedures because of specific religious and cultural prejudices. Later, if a problem happens to the patient, we are the first to be questioned.”*

#### Sub/subcategory 2: environmental factors

The study participants’ perceptions of additional stressors in handling pre-hospital emergencies were included in the subcategory. This subcategory included codes related to the possibility of unforeseen and unpredictable events at the accident scene, the lack of space in the ambulance for emergency procedures, being sent on missions in unfavorable weather or environmental conditions, the difficulty of accessing patients due to traffic and crowding at the scene, the inability to transfer practical training to the clinical setting because of the stressful nature of the accident scene, the transfer of stress from bystanders, the lack of police presence at the scene, the loss of equipment and tools at the scene, the risk of explosions caused by flammable materials, the interference of bystanders in the provision of pre-hospital care, and a high number of injuries in the accident. In this regard, the following are quotes taken from the interviews conducted with the study participants:

- P3: *" There are situations where the patient's condition cannot be predicted and we really do not know what will happen. In this situation, considerable stress endures until the patient is admitted to the hospital. It is not predictable.”*
- P2:* "The scene of the accident was so crowded and uncontrollable that we forgot to inject the epinephrine drug that we had to administer to the patient in cardiopulmonary resuscitation."*

### Category 4: the complex and multifaceted context of stressful pre-hospital emergencies

This category focuses on the experiences of EMTs in dealing with the stressors of pre-hospital emergencies. Participants identified various factors and triggers that contributed to stress in these situations. This category was further divided into three sub-categories: stress in dealing with pediatric emergencies, stress in dealing with cardiac emergencies, and stress in dealing with gynecological emergencies.

#### Subcategory 1,2: stressors of pediatric and gynecological emergencies

During the interviews, the EMTs highlighted several challenges associated with pediatric emergencies. One significant problem is the difficulty in accurately diagnosing the current illness in pediatric patients. EMTs often face the serious challenge of rapid and early diagnosis of a patient's condition. Other stressors included the following.

- Difficulty in establishing proper communication with children
- Lower frequency of pediatric emergencies compared to adults
- Differences in the physiology and anatomy of children
- Insufficient information about the disease provided by companions
- Current condition of the patient and the possibility of injury during emergency procedures
- Lack of agreement on protocols and training for data collection
- Gap between theory and practice in the implementation of procedures in pediatric emergencies

For instance, one technician expressed their viewpoint on the stressors of obstetrical and gynecological emergencies:

- P4: *"As soon as the operator informs us of a gynecological and obstetric emergency, we get stressed, especially in cases where the route is far or it happens at night. Sometimes we really can't do anything for the patient, for example, the baby gets stuck in the birth canal."*

#### Subcategory 3: stressors of cardiac emergencies

In the context of cardiac emergencies, technicians have identified the lack of up-to-date and sufficient equipment as a significant stressor. One participant shared their perspective on this issue.

- P3: *"We do not have a telecardiology device in the pre-hospital emergency room. Well, we do not have sufficient knowledge of (Electrocardiogram) EKG, sometimes we mistakenly did not activate the cardiac code for the hospital because we thought that the patient had no EKG problem, in the event that the patient went straight to the lab as soon as he entered the hospital and received the correct diagnosis."*

These quotes illustrate the challenges and stressors faced by EMTs when dealing with pediatric, gynecological, and cardiac emergencies. Understanding these stressors is crucial for developing strategies to support and enhance EMT performance in high-pressure situations.

## Discussion

The initial goal of this study was to examine PTSD in EMTs. The findings of this study showed that approximately 20.1% of technicians displayed symptoms that met PTSD criteria. This prevalence is comparatively high compared to other studies in the same field [39, 40]. Univariate and multivariate linear regression analyses were employed to predict the relationship between potential risk and protective factors that might impact technicians’ PTSD scores. The results of the univariate regression analysis demonstrated that, among the various factors considered, the number of shifts worked per month had the greatest influence on technicians' PTSD scores. Specifically, the likelihood of experiencing PTSD symptoms increased by 0.8% for each additional shift per month. This finding aligns with research by Elle Nguyen and colleagues, who identified sleep disorders as a primary cause of PTSD among EMTs [41]. In the qualitative phase of our study, technicians also reported frequent sleep disturbances as a significant stressor. Additionally, the stress associated with managing pre-hospital emergencies leads to delayed initiation of nighttime sleep and reduced sleep quality. These qualitative findings confirm the quantitative results, where irregular 24-h shifts were found to be the most significant and predictable factor influencing the onset and development of PTSD among the technicians. Overall, the findings of this study shed light on the high prevalence of PTSD in EMTs and emphasize the critical role of factors such as the number of shifts worked per month and sleep disturbances in contributing to this mental health condition. Managing these risk factors and implementing interventions to improve sleep quality and manage work schedules may be crucial to mitigating PTSD symptoms in EMTs.

In this qualitative study, one of the main categories extracted was the overall impact of the stress crisis on EMTs, which itself consisted of three sub-categories: physical-physical effects, mental-psychological-mental effects, and occupational-functional effects. Under the physical effects sub-category, EMTs reported experiencing various symptoms due to the stress of dealing with prehospital emergencies. These symptoms include increased heart rate, headache and nausea, heavy sweating, spasms and muscle cramps, sleep disturbances caused by stressful events, and excessive fatigue. Research findings have also indicated a significant relationship between the stress of dealing with prehospital emergencies and the occurrence of severe physical disorders among EMTs. For instance, studies have shown that EMTs are at a higher risk of developing acute and chronic back pain [42]. Additionally, a systematic review by Lentz et al. revealed that increased exposure to prehospital emergencies and the physical demands of the job led to musculoskeletal injuries among emergency care providers [43]. Overall, the findings highlight the physical and occupational consequences of the stress crisis faced by EMTs and emphasize the importance of addressing these issues to ensure the well-being of healthcare professionals.

In a study conducted by Leblanc et al., EMTs often experienced mental conflict caused by stress, which had a significant impact on their performance [13]. Frequent exposure to acute and stressful situations as part of daily tasks can make EMTs vulnerable in areas such as documenting information and clinical competence. One of the sub-categories mentioned by technicians in relation to the effects of stress is the spiritual-psychological-mental aspect. Technicians reported feelings of regret about their career choices, concerns about accidents happening to themselves and their families, and obsessive thoughts about perceived negligence in their performance. They also mentioned reduced self-confidence in sensitive situations and constant anxiety about the occurrence of accidents. Occupational-functional effects were another subcategory mentioned by the technicians. These effects include a decrease in performance and competence in their role as EMTs, as well as difficulty in remembering traumatic events and scenes related to their work (2.25 average). A quantitative study of post-traumatic stress disorder (PTSD) found that the dimension of negative changes in mood or cognition had the highest average score (7.42) among EMTs. This indicates that EMTs experience significant negative emotional and cognitive effects when faced with stressful prehospital emergencies. These effects can affect all aspects of life. Shepherd et al. also found that EMTs with PTSD symptoms struggled to moderate their negative emotions and cognitive changes. This further highlights the impact of these dimensions on the occurrence of PTSD [44]. Overall, this study suggests that EMTs face various spiritual, psychological, mental, and occupational challenges due to the stress encountered during pre-hospital emergencies. These challenges can affect their performance and well-being, highlighting the importance of addressing the psychological well-being of EMTs to enhance their overall effectiveness in the field.

In the present study, one of the subcategories extracted was the complex and multifaceted nature of prehospital emergency stress. This subcategory includes stress related to pediatric, cardiac, and gynecologic emergencies. Technicians have identified these three stressors as common causes of pre-hospital stress. The technicians specifically cited several reasons that explain the stressful nature of pediatric emergencies. Poor communication, poor diagnosis, and limited exposure to aggressive interventions were identified as contributing factors. These factors can cause information to be forgotten and skills decline over time. Jeruzal et al. conducted a qualitative study and reported similar finding [44]. Their research showed that technicians find pediatric emergencies stressful because of their rarity and high-risk potential in the prehospital system. Lack of verbal communication with infants and children, as well as the complex clinical conditions that require the use of advanced equipment such as ventilators and tracheostomies, also contribute to the burden on technicians. In our study, a lack of consensus on data collection protocols in pediatric emergencies was cited as an additional source of stress for technicians. This inconsistency in protocols can make it difficult for technicians to effectively manage pediatric emergencies. Drews et al. highlighted a specific protocol, the Glasgow Coma Scale, which only had agreement for collecting information related to consciousness level in about 61% of cases between pre-hospital emergency protocols and trauma department protocols [45]. In trauma cases, this agreement dropped to less than 52%, which posed a problem for EMTs. Overall, the complex and multifaceted nature of pre-hospital emergency stress, particularly in the context of pediatric emergencies, highlights the challenges faced by technicians in this field. Adequate training, communication and standardized protocols are essential to reduce stress and ensure optimal patient care.

Cardiac emergencies are stressful for both patients and medical staff. Several factors contribute to this stress response. Reasons for the stressful nature of cardiac emergencies include lack of specialized cardiac centers that can refer patients more quickly, lack of two experienced technicians to handle cardiac emergencies, physical strain and fatigue, and inadequate guidelines for resuscitation, among others, according to technicians, inefficiency of equipment due to additional costs, difficulty in transporting cardiac patients, incomplete knowledge of defibrillator use and ECG interpretation. Furthermore, Dehghan-Nayeri et al. reported that outdated respiratory cardiac resuscitation devices also represent a stressor causing cardiac emergencies in the prehospital system [46].

Emergency obstetrics is a stressful emergency situation that technicians must address. Lack of experience and training in dealing with obstetric emergencies and not using female technicians in prehospital emergencies are among the most significant causes of stress from the perspective of technicians. Owing to a lack of practical training on real patients and sufficient experience, technicians often experience significant stress during obstetric emergencies. Persson et al. recommends that due to inadequate preparation and knowledge, nurses receive training in simulated environments using previously developed scenarios to help them deal with emergency situations [47].

Our study found that environmental factors, such as the interference and involvement of bystanders in providing pre-hospital care, were identified as stressors for EMTs. According to the technicians, these factors disrupted necessary care for patients [48]. Additionally, technicians reported that the lack of timely and appropriate care due to a chaotic scene and the scarcity of suitable parking spaces for ambulances sometimes led to conflicts and violence with 115 personnel at the scene of the accident. Weaver et al. found that over 50% of occupational injuries and violence against EMTs occurred at the scene of the accident and caused the most stress [49].

One of the categories identified in this study was "missing link in communication networks in incident management," which comprised three subcategories: inter-, intra-, and extra-organizational. In terms of the first subcategory, participants reported that pre-hospital emergency management was affected by stressors such as delays in the rapid transfer of patients due to concerns about being fined by the police for speeding, as well as speeding. According to Murray et al., only 1% of patients who did not use sirens and alarms during ambulance transfers had worse outcomes [50]. Watanabe also pointed out that the use of sirens and alarms by technicians when transporting patients to hospitals nearly tripled the number of accidents [51]. Therefore, there is still much debate about increased speed and the use of warning devices in ambulances when transferring patients and their impact on technicians and patients.

One of the limitations of our study in our research community was that all emergency medical technician technicians working in the 115-emergency department of Hamadan province were male. This made it impossible to study and examine the independent variables on female operational technicians and compare the results with male technicians.

## Conclusion

Work stress can vary depending on the nature of the workplace, and for pre-hospital emergencies, dealing with different types of emergencies can cause stress. The quantitative and qualitative results of this study show that modifying several factors reduces technicians' stress, such as reducing working hours, providing ongoing training education, increasing the number of facilities and staff, insuring ambulance equipment, not hiring clinically unqualified staff, using psychologists in emergency situations, assisting staff with legal issues and contracts, recruiting midwives in emergency situations, updates to procedures and guidelines for pre-hospital systems, coordination between EMTs and police, and the use of advanced technologies can help reduce stress and manage stressors when faced with a prehospital emergency. future research could explore the effectiveness of specific interventions aimed at mitigating stress and managing stressors encountered during prehospital emergencies. Additionally, longitudinal studies could be conducted to track changes in EMTs' mental health over time and evaluate the long-term impact of interventions.

## Data Availability

The datasets used and/or analysed during the current study are available from the corresponding author on reasonable request.

## Abbreviations

EMTs: Emergency Medical Technicians
PCL-5: The PTSD Checklist for DSM-5
PTSD: Post Traumatic Stress Disorder
EKG: Electrocardiogram
